# ATP-mediated Events in Peritubular Cells Contribute to Sterile Testicular Inflammation

**DOI:** 10.1038/s41598-018-19624-3

**Published:** 2018-01-23

**Authors:** Lena Walenta, David Fleck, Thomas Fröhlich, Hendrik von Eysmondt, Georg J. Arnold, Jennifer Spehr, J. Ullrich Schwarzer, Frank-Michael Köhn, Marc Spehr, Artur Mayerhofer

**Affiliations:** 1 Biomedical Center Munich (BMC), Cell Biology, Anatomy III, Ludwig-Maximilians-Universität München, 82152 Planegg-Martinsried, Germany; 2 Department of Chemosensation, Institute for Biology II, RWTH Aachen University, 52074 Aachen, Germany; 3 Laboratory for Functional Genome Analysis LAFUGA, Gene Center, Ludwig-Maximilians-Universität München, 81377 Munich, Germany; 4 Andrology Center, 81241 Munich, Germany; 5 Andrologicum, 80331 Munich, Germany

## Abstract

Peritubular myoid cells, which form the walls of seminiferous tubules in the testis, are functionally unexplored. While they transport sperm and contribute to the spermatogonial stem cell niche, specifically their emerging role in the immune surveillance of the testis and in male infertility remains to be studied. Recently, cytokine production and activation of Toll-like receptors (TLRs) were uncovered in cultured peritubular cells. We now show that human peritubular cells express purinergic receptors P2RX4 and P2RX7, which are functionally linked to TLRs, with P2RX4 being the prevalent ATP-gated ion channel. Subsequent ATP treatment of cultured peritubular cells resulted in up-regulated (pro-)inflammatory cytokine expression and secretion, while characteristic peritubular proteins, that is smooth muscle cell markers and extracellular matrix molecules, decreased. These findings indicate that extracellular ATP may act as danger molecule on peritubular cells, able to promote inflammatory responses in the testicular environment.

## Introduction

Male infertility is common and in a considerable number of cases the underlying causes are not known^1,2^. In infertile men, impairments of spermatogenesis are typically paralleled by alterations of testicular morphology. Common changes include fibrotic thickening of the tubular wall, and accumulation of macrophages and mast cells in both the testicular interstitial area and the tubular wall^3–6^. These alterations point to a form of sterile inflammation in the testes, specifically prevalent in the tubular wall, which is formed by peritubular cells and extracellular matrix (ECM).

Peritubular myoid cells are smooth muscle-like cells known for their contractile abilities that are of utmost importance for sperm transport^7,8^. Previous studies, including proteomic and secretomic analyses, revealed that these human testicular peritubular cells (HTPCs) secrete ECM components and act as paracrine signalling cells^9^. Intriguingly, they also secrete immunoregulatory factors^10^. Recently, Toll-like receptors (TLRs) as functional key regulators of innate immune responses were identified in HTPCs^11^. It became evident that ligands like Pam3CysSerLys4 (PAM) or lipopolysaccharide (LPS) are able to activate TLR2/4 on peritubular cells. In addition, TLR2/4 was also targeted by the small ECM molecule biglycan in the same way as previously found in macrophages^12^. Biglycan-induced TLR signalling triggered an immune response including pro-inflammatory cytokine production and secretion^13,14^.

In this context, simultaneous activation of TLR2/4 and the purinergic receptor isoforms P2RX4 and P2RX7 by biglycan has been discovered^15^. Both, P2RX4 and P2RX7, represent members of a family of ligand-gated ion channels that are activated by ATP at either relatively low (P2X4; EC_50_~1–10 µM) or substantially increased (P2X7; EC_50_~100–300 µM) concentrations^16^. In the testis, potential origins of extracellular ATP are infiltrating immune cells like mast cells and macrophages, as well as Sertoli cells^17,18^. Both cell types reside in the immediate vicinity of peritubular cells^3,19,20^. Thus, we hypothesized that ATP may act as a danger molecule in the testes in the context of sterile inflammation and may promote inflammatory responses in HTPCs. We explored this possibility in a human-focused approach.

## Results

### Peritubular cells express the purinergic receptors P2RX4 and P2RX7

**Figure 1:**
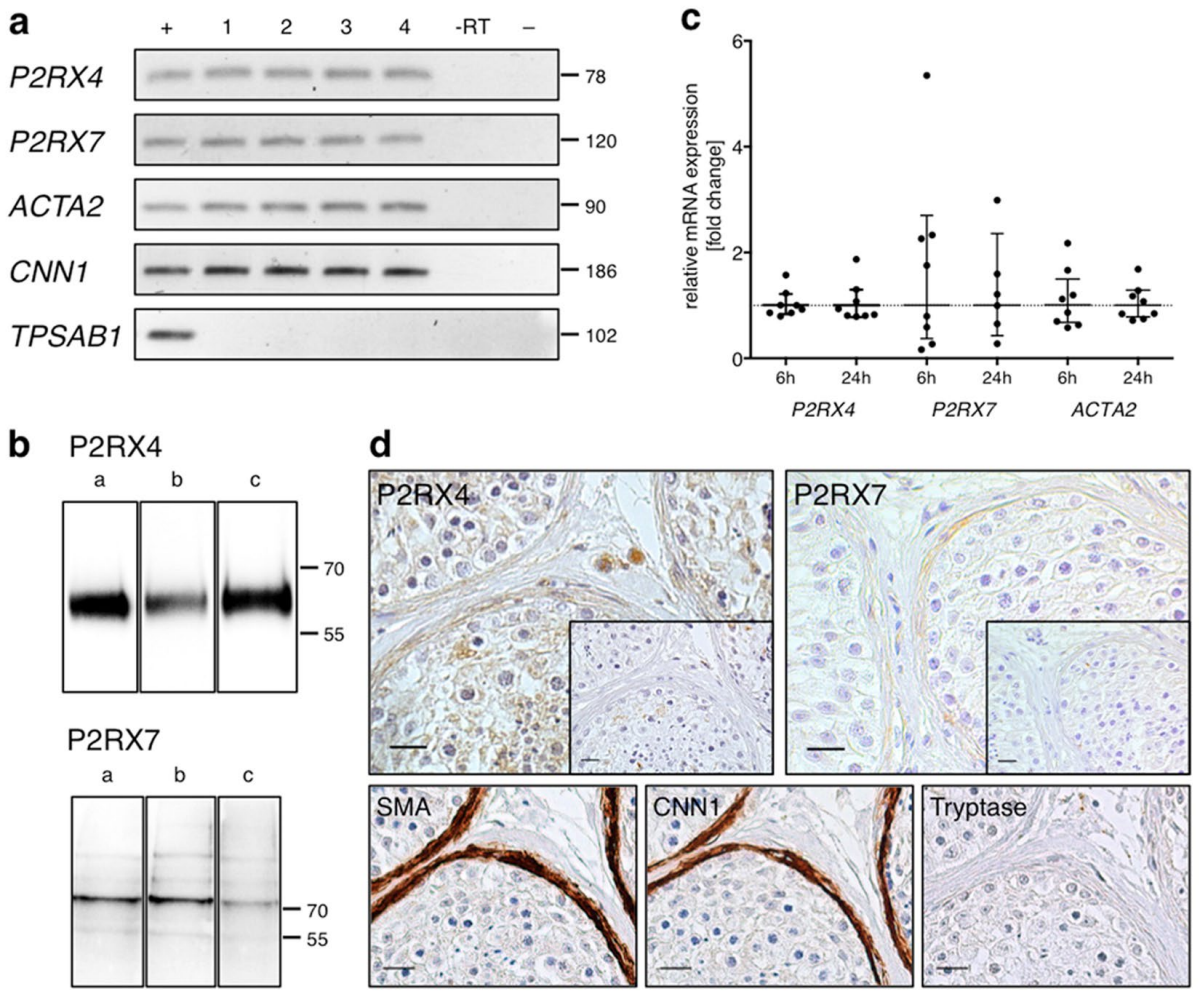
Expression of purinoceptors P2RX4 and P2RX7 in peritubular cells. (**a**) Expression of *P2RX4* and *P2RX7* mRNA was revealed in HTPCs stemming from four individual patients (1–4) and in the human testis (+). Patient-derived HTPCs were additionally screened for the presence of smooth muscle cell markers *ACTA2* and *CNN1* and absence of the mast cell marker *TPSAB1*. Negative controls: non-reverse transcription control (−RT), non-template control (−). (**b**) Immunoblotting confirmed P2RX4 and P2RX7 expression in HTPCs from three different patients (a–c). Note that gel/blot images were cropped and full-length gels/blots are presented in Supplementary Fig. 5. (**c**) Basal *P2RX4* (n = 8) and *P2RX7* (n = 8/6) receptor mRNA levels at 6 h and 24 h varied between cells derived from individual patients, but also *ACTA*2 levels (n = 8) did not remain constant. Data are geometric mean with 95% confidence interval. (**d**) P2RX4 expression was detected in peritubular cells, germ cells and interstitial tissue, while P2RX7 expression was found in peritubular cells and vessels (not shown) solely. P2RX4 expression in the tubular wall overlapped with SMA and CNN1 expression and absence of tryptase staining in consecutive sections. Insets: Negative controls (pre-adsorption for P2RX4, omission of primary antibody for P2RX7); Bars = 20 µm.

Expression of purinoceptor subtypes P2RX4 and P2RX7 in cultured HTPCs of different patients was demonstrated on both, transcript and protein level (Fig. 1a,b). All individual donor-derived cells expressed typical smooth muscle cell marker transcripts A*CTA2* (Actin, aortic smooth muscle) and calponin (*CNN1*)^5,6,21^, but lacked expression of the mast cell marker tryptase (*TPSAB1*, Fig. 1a)^3^. HTPC cultures were additionally analysed by CNN1 immunofluorescence staining (Supplementary Fig. 1) to confirm marker expression and purity of the cultured cells. Basal *P2RX4* and *P2RX7* receptor mRNA expression levels, but also *ACTA2* expression levels varied between cultured cells from individual patients (Fig. 1c). In human testicular sections (Fig. 1d) P2RX4 was detected in peritubular cells, but also in germ cells and in the interstitial tissue by immunohistochemistry. P2RX7 expression in the human testis was confined to peritubular cells and endothelial cells of blood vessels (not shown). Staining of consecutive sections showed that immunoreactive peritubular cells expressed smooth muscle actin (SMA) and CNN1. In fibrotically thickened walls of seminiferous tubules, in which impairment of spermatogenesis was evident, P2RX4 and P2RX7 were readily observed (Supplementary Fig. 2a,b). The presence of mast cells as a possible source of extracellular ATP in the immediate vicinity of the tubular wall, and therefore to the purinoceptors, was confirmed (Supplementary Fig. 2c–f).

### HTPCs are sensitive to purinergic stimulation *in vitro*

**Figure 2:**
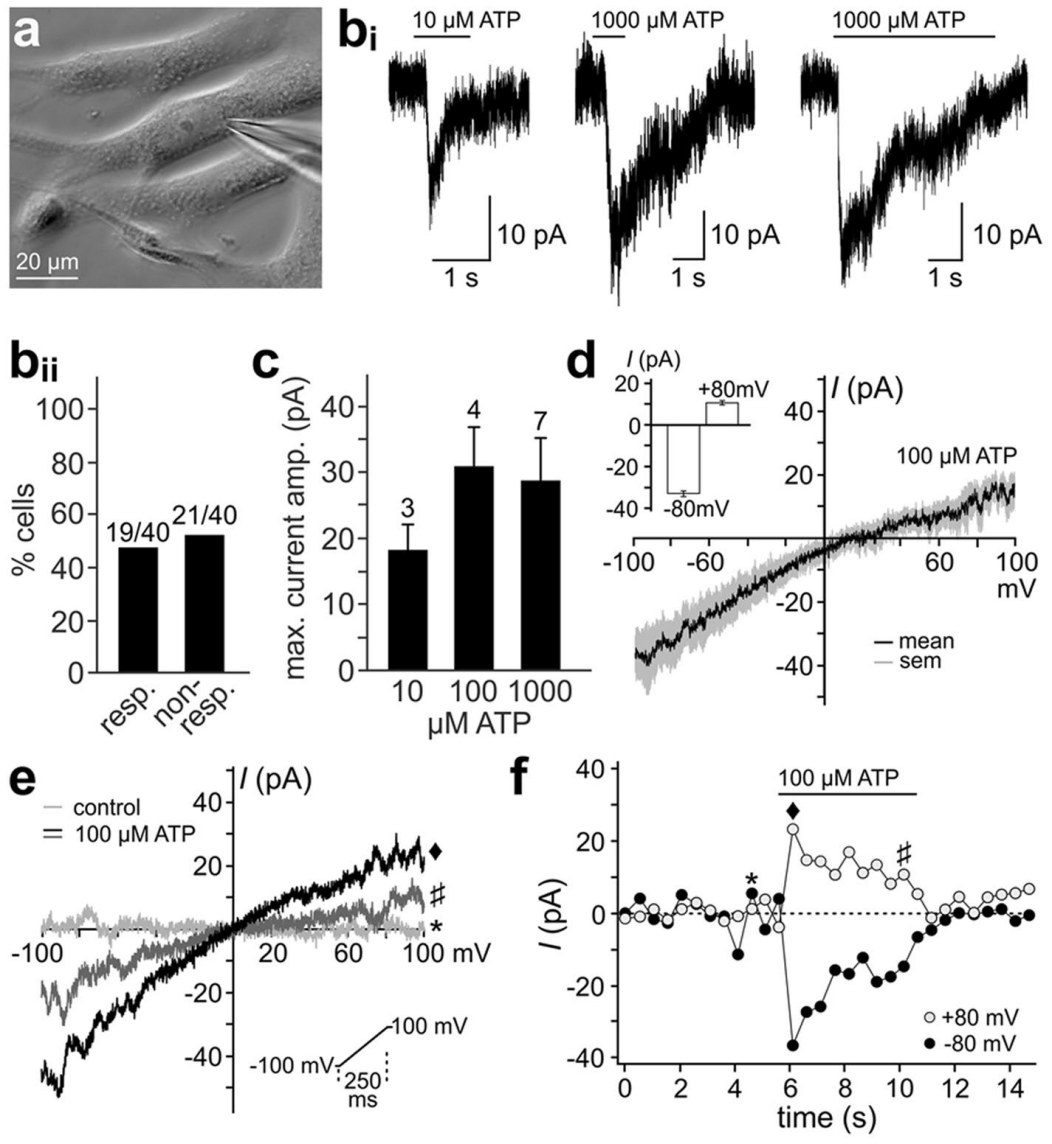
Extracellular ATP stimulates HTPCs. (**a**) Phase-contrast micrograph depicting cultured HTPCs. A single cell is targeted by a patch pipette. (**b**_**i**_) Original current traces from representative whole-cell patch-clamp recordings (*V*_hold_ = −40 mV; **S**_**1**_, **S**_**4**_) from cultured HTPC challenged with ATP at increasing concentrations (10, 1,000 µM) and exposure durations (1 s, left and middle; 5 s right). Note that prolonged stimulation clearly revealed current desensitization. For clarity, currents were smoothed according to a ‘box 7’ algorithm (Igor Pro software) (**b**_**ii**_) Bar graph quantifying the percentage of ATP-sensitive HTPCs. (**c**) Quantification of recordings as shown in (**b**). Bar chart depicting peak current amplitude measurements (mean ± SEM) in response to variable [ATP]_ex_ (10, 100 and 1,000 µM). Numbers of experiments are indicated above bars. (**d**) Average maximal *I–V* curve (black trace) in response to 100 µM ATP (n = 4). Grey shadows indicate SEM. Inset shows mean currents at −80 mV and +80 mV, revealing substantial inward rectification. Representative current–voltage relationships (**e**) and current time course (**f**) in response to 100 µM ATP. Inset (**e**): Command voltage ramp, repeated at 2 Hz. (**f**) Representative plots of current measurements at −80 mV (black dots) and +80 mV (empty circles), respectively, over time. When challenged with 100 µM ATP, a fast, but relatively small inward current develops and shows a transient peak followed by apparent desensitization.

To address whether HTPCs could be part of a paracrine purinergic signalling network^22^, we performed whole-cell patch-clamp recordings from cultured cells (Fig. 2a). HTPCs exhibited an average membrane capacitance (*C*_mem_) of 27.5 ± 8.3 pF. When cells were exposed to ATP (10–1,000 µM) at a negative holding potential (*V*_hold_ = −80 mV), we consistently recorded a fast-activating inward current (*I*_ATP_) in 47.5% of all HTPCs, whereas no such current was recorded in a similarly large fraction of cells (52.5%; Fig. 2b_i-ii_). Upon prolonged exposure (5 s), the current saturated and then monotonically declined in presence of the stimulus (Fig. 2b_i_). Next, we asked whether ATP sensitivity is dose-dependent. At increasing extracellular ATP concentrations ([ATP]_ex_) of 10, 100 and 1,000 µM, HTPCs exhibited dose-dependent *I*_ATP_ amplitudes with apparent saturation at [ATP]_ex_ ≥ 100 µM (Fig. 2c). In sharp contrast to previous observations in mouse spermatogonia^23^, the relatively small, desensitizing inward currents initiated by lower [ATP]_ex_ (10–100 µM) did not change in amplitude, kinetics or desensitization rate upon exposure to high stimulus concentrations (1,000 µM). To examine the current–voltage relationship of *I*_ATP_, we measured I–V curves at a rate of 2 Hz before, during and after stimulation with saturating [ATP]_ex_ (100 µM; Fig. 2d–f). Currents reversed at approximately 0 mV and exhibited substantial inward rectification (Fig. 2d–f). The above properties all indicate that P2RX7 does not serve as the predominant ATP receptor in HTPCs^24–26^. Rather, our data suggest that cultured HTPCs express one or more purinergic receptor isoform(s) characterized by relatively high ATP sensitivity.

### HTPC purinergic signals are likely mediated by P2RX4 receptors

**Figure 3:**
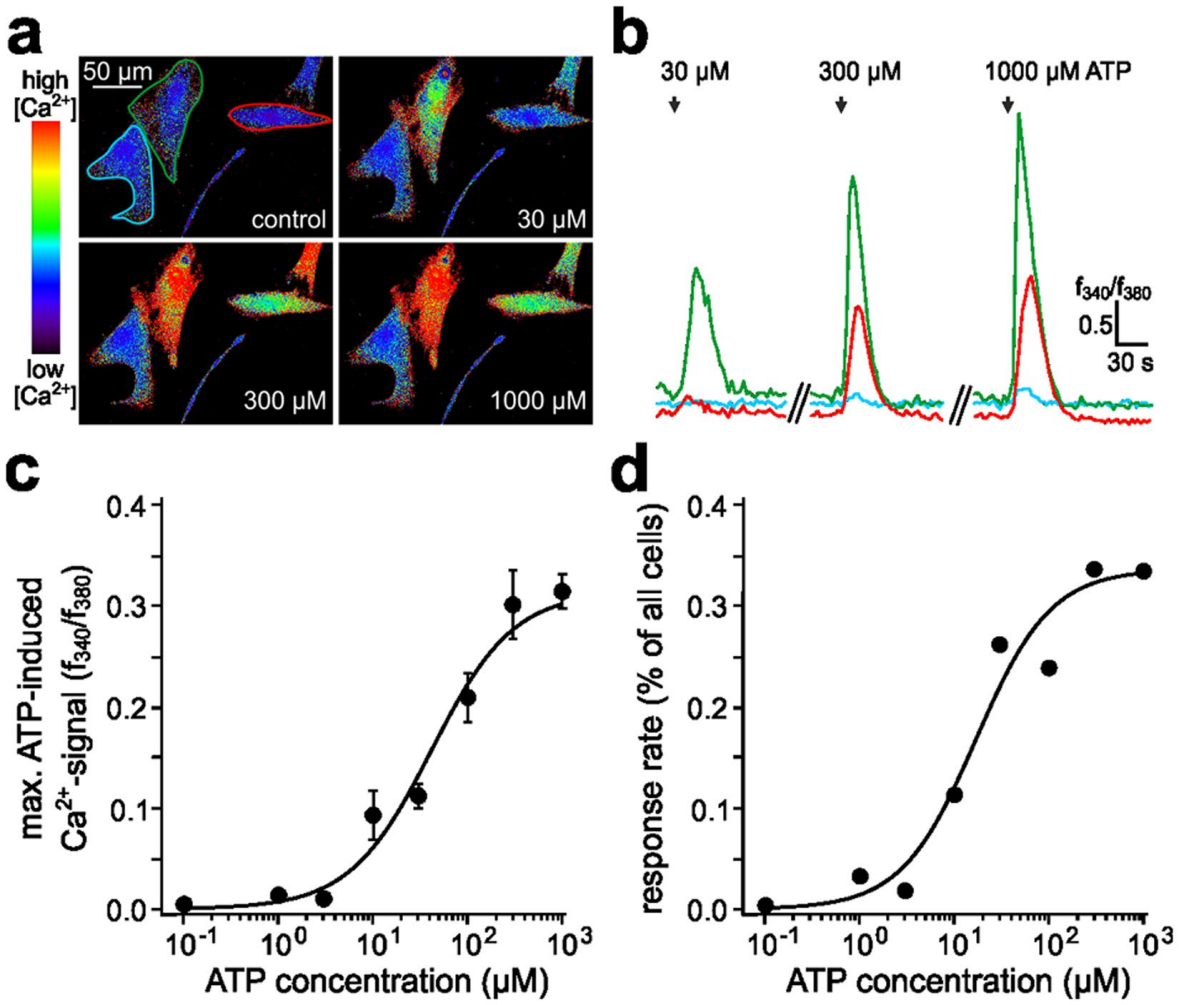
ATP-dependent Ca^2+^ mobilization in HTPCs. (**a** and **b**) HTPC [Ca^2+^]_c_ signals in response to increasing ATP concentrations (30, 300 and 1,000 µM; 1 s) were monitored by ratiometric fluorescence imaging in fura-2/AM-loaded cells. (**a**) Pseudocolour single frame images illustrate relative [Ca^2+^]_c_ at different time points (rainbow 256 colourmap; blue = low Ca^2+^/red = high Ca^2+^). (**b**) Original traces depict the integrated fluorescence ratio f_340_/f_380_ of representative cells in user-defined regions of interest (ROIs; colour as in (**a**)) as a function of time. Note the discontinuous ordinate (***//***). (**c** and **d**) Average dose-response (**c**) and dose-‘recruitment’ (**d**) curves depict peak elevations in [Ca^2+^]_c_ (c) and the percentage of responsive HTPCs (**d**) upon exposure to increasing ATP concentrations, ranging from 0.1 µM to 1,000 µM. The threshold concentration for activation is ~3 µM, half-maximal activation/recruitment (EC_50_) is observed at 41 µM (**c**) and 15 µM (**d**), respectively, and saturated signals are induced by ATP concentrations of ≥300 µM. Sigmoid dose-response curves were calculated using the Hill equation. Individual data points in (**c**) show means ± SEM (n = 36–158).

To increase throughput, we next opted to analyse ATP sensitivity using ratiometric Ca^2+^ imaging of HTPC populations. For recording of cytosolic Ca^2+^ concentrations [Ca^2+^]_c_, HTPCs were loaded with fura-2/AM and briefly (3 s) exposed to increasing doses of ATP (0.1–1,000 µM; Fig. 3a). In many cells (33.5%), ATP stimulation triggered an immediate and robust [Ca^2+^]_c_ signal that decayed after stimulus cessation (Fig. 3b). It is likely that the size of this responsive population is an underestimate since many of the remaining HTPCs (66.5%) were precluded from analysis because of spontaneous activity (see Material and Methods). When both peak [Ca^2+^]_c_ signals (Fig. 3c) and response frequencies (Fig. 3d) were plotted as a function of stimulus concentration, ATP consistently induced dose-dependent responses with a shared activation threshold of ~3 µM. The linear dynamic range of the dose-response curves (Fig. 3c,d) spanned ~2 logarithmic units (EC_50_ = 41 µM) and Ca^2+^ signals saturated at ~300 µM [ATP]. Since P2RX7, compared to other family members, exhibits substantially reduced ATP sensitivity [EC_50_ ≥ 300 µM]^25,27,28^, these results also point to expression of purinoceptor isoform(s) that display high ATP sensitivity.

**Figure 4:**
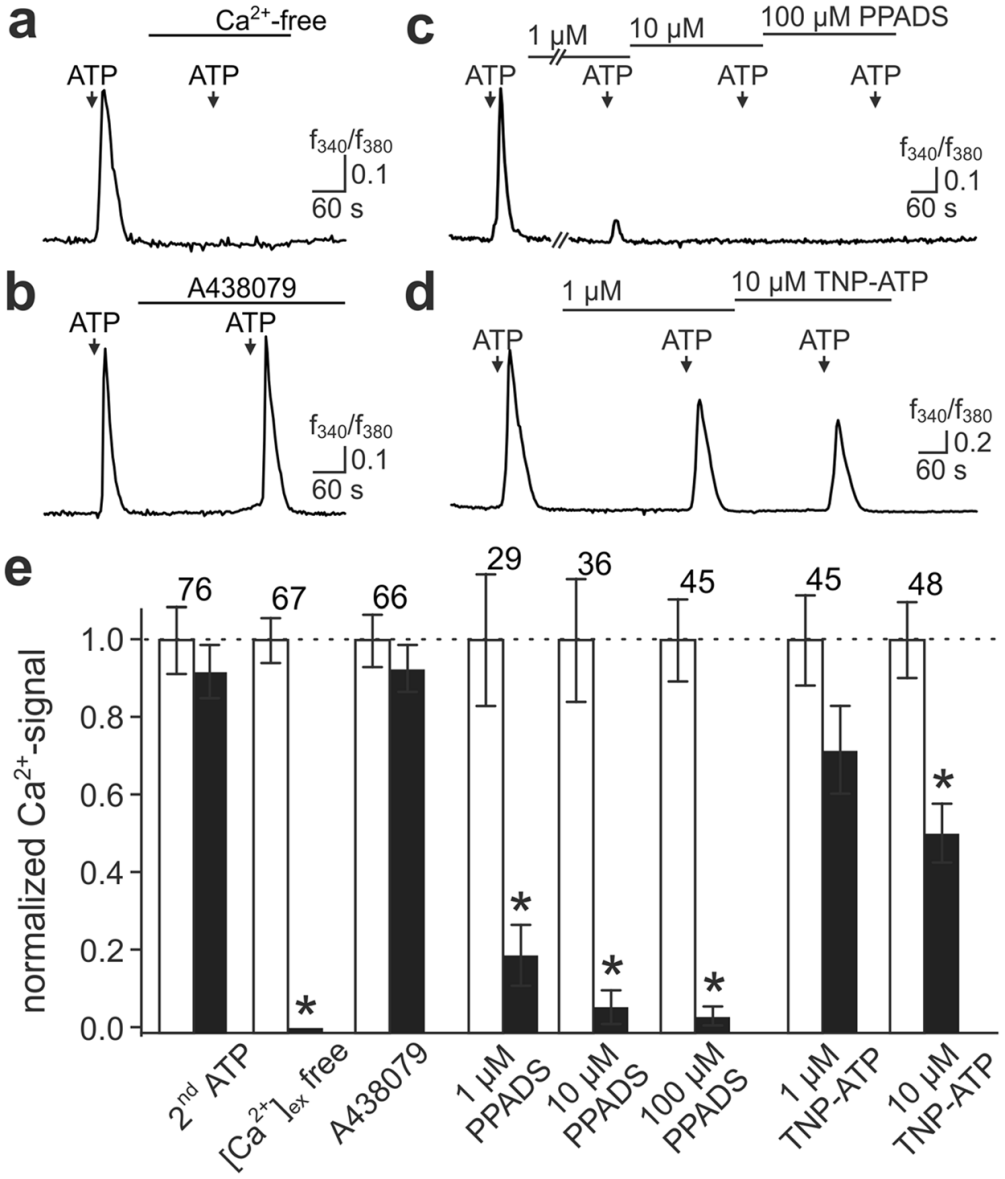
Pharmacological signature of ATP-dependent HTPC Ca^2+^ signals. (**a**–**d**) Representative original traces (f_340_/f_380_ *versus* time) of [Ca^2+^]_c_ transients recorded from individual HTPCs challenged with ATP (1000 µM (**a**,**b**); 30 µM (**c**,**d**); 5 s; arrows) under control conditions and during different biophysical/pharmacological treatments. Signals are abolished in absence of external Ca^2+^ (**a**), essentially unaffected by the P2RX7 receptor antagonist A438079 (10 µM (**b**)), and strongly or partly reduced in presence of PPADS (**c**) or TNP-ATP (**d**), respectively. Treatment duration (pre-incubation) is indicated by horizontal bars. (**e**) Bar chart quantifying the effects of different conditions on [Ca^2+^]_c_ response amplitudes (black bars). Data are means ± SEM, normalized to control conditions (i.e., prior stimulation; white bars). Numbers of cells are indicated above bars. Asterisks (*) denote statistical significance, *p* < 0.05 (paired *t*-test).

Next, we sought to identify the involved P2X receptor isoforms by pharmacological fingerprinting of ATP-induced [Ca^2+^]_c_ signals (Fig. 4). First, HTPCs were exposed repeatedly to saturating ATP concentrations (1,000 µM) in presence of standard (1 mM; **S**_**1**_) *versus* reduced (165 nM; **S**_**2**_) free extracellular [Ca^2+^]. Notably, we never observed ATP-induced [Ca^2+^]_c_ elevations when extracellular [Ca^2+^] was reduced (Fig. 4a,e). This finding shows that, first, Ca^2+^ influx from the extracellular medium is necessary for ATP-mediated responses and that, second, P2Y receptor-dependent depletion of intracellular stores does not serve a primary function in HTPC purinergic signalling. Next, stimulations were carried out in presence of the selective P2RX7 receptor antagonist A438079 (10 µM), which blocks human P2RX7 at nanomolar concentrations^26^ but, in HTPCs, failed to affect ATP-mediated signals (Fig. 4b,e).

In a third series of pharmacological tests, HTPCs were exposed to a non-saturating ATP concentration (30 µM) in presence of different concentrations of either PPADS (Pyridoxal phosphate-6-azo(benzene-2,4-disulfonic acid); Fig. 4c,e) or TNP-ATP (2′,3′-O-(2,4,6-Trinitrophenyl)adenosine-5′-triphosphate; Fig. 4d,e). Both agents are partially selective P2X receptor antagonists that inhibit human P2RX isoforms with variable efficacy. In HTPCs, ATP-induced [Ca^2+^]_c_ signals were clearly affected by PPADS (1 µM) and essentially abolished by exposure to high drug concentrations (100 µM; Fig. 4c,e). By contrast, only elevated TNP-ATP concentrations (10 µM) significantly diminished ATP-mediated responses (Fig. 4d,e). Human P2RX1–3 and P2RX5 are blocked by nanomolar concentrations of both PPADS and TNP-ATP^29^. By contrast, human P2RX4 is considerably less sensitive to both drugs, displaying IC_50_ values in the mid-micromolar range^29,30^. Together, our pharmacological data are thus most consistent with functional expression of homomeric P2RX4 receptors in cultured HTPCs. This interpretation is further supported by the electrophysiological signature (desensitization with time constants of up to several seconds; pronounced inward rectification) and sensitivity (saturation at [ATP]_ex_ ≥ 100 µM) of *I*_ATP_ (Fig. 2).

### ATP mediates changes of peritubular cell characteristics and fosters up-regulation of inflammatory molecules

HTPCs are smooth muscle-like cells, which also secrete extracellular matrix, contribute to the spermatogonial stem cell niche and exhibit immunoregulatory functions^6^. To assess the impact of ATP on these characteristic features of HTPCs, three different strategies were applied: evaluation of changes in transcript levels, regulation of secreted cytokines in the supernatant and a whole cell proteomic approach. Cell viability during treatment was monitored via cell number and confluence determination in a live cell imaging system and additionally by cytotoxicity measurement via lactate dehydrogenase (LDH) release. Results revealed that cellular viability was not negatively influenced by ATP (Supplementary Fig. 3).

**Figure 5:**
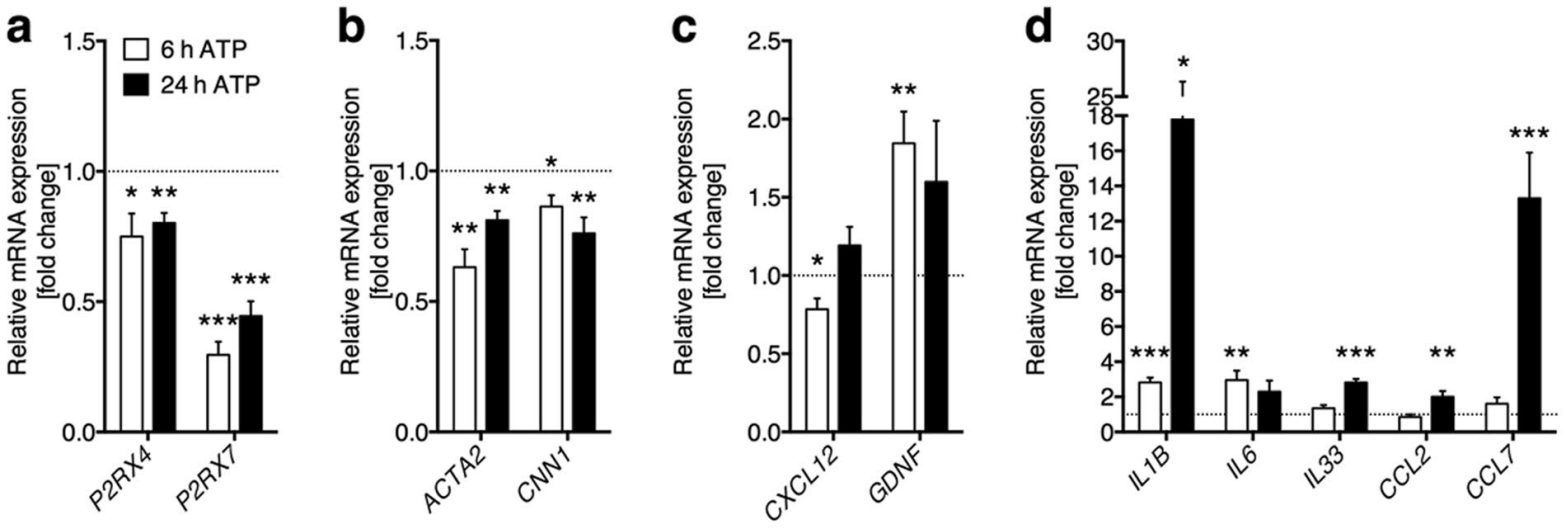
ATP treatment affected characteristic peritubular markers and inflammation-associated genes. (**a**) After ATP treatment of HTPCs *P2RX4* (n = 8) and *P2RX7* (n = 8/6) mRNA levels were significantly down-regulated. (**b**) mRNA levels of smooth muscle cell markers *ACTA2* (n = 8) and *CNN1* (n = 8) were significantly decreased by ATP treatment. (**c**) Stem cell niche regulatory factors *CXCL12* (n = 8) and *GDNF* (n = 8/6) mRNAs were marginally influenced in a time-dependent manner. (**d**) Inflammation-associated genes *IL1B* (n = 8/5), *IL6* (n = 8), *IL33* (n = 8), *CCL2* (n = 8) and *CCL7* (n = 8/6) mRNA levels were increased after ATP treatment. Data are means ± SEM after 6 h and 24 h, normalized to control conditions. Asterisks denote statistical significance, *p < 0.05, **p < 0.01, ***p < 0.001 (one-sample *t*-test).

ATP treatment resulted in a distinct decrease of purinoceptor mRNA expression after 6 h and 24 h (Fig. 5a). Characteristic smooth muscle cell markers (*ACTA2*, *CNN1*) were similarly decreased (Fig. 5b). ATP affected stem-cell niche regulatory factors (*CXCL12*, *GDNF*) only marginally, but in a time-dependent fashion (Fig. 5c). However, a panel of inflammation-associated genes showed moderately (*IL6*, *IL33*, *CCL2*) to substantially (*IL1B*, *CCL7*; Fig. 5d) increased expression. Yet, there was a different time-dependence in the mRNA elevation. While most transcript levels rose until 24 h, *IL6* clearly reached a peak before 24 h. These changes suggest that ATP induces a switch from the smooth muscle cell-like phenotype and towards an immune-regulatory phenotype as it has been observed in vascular smooth muscle cells^31^.

**Figure 6:**
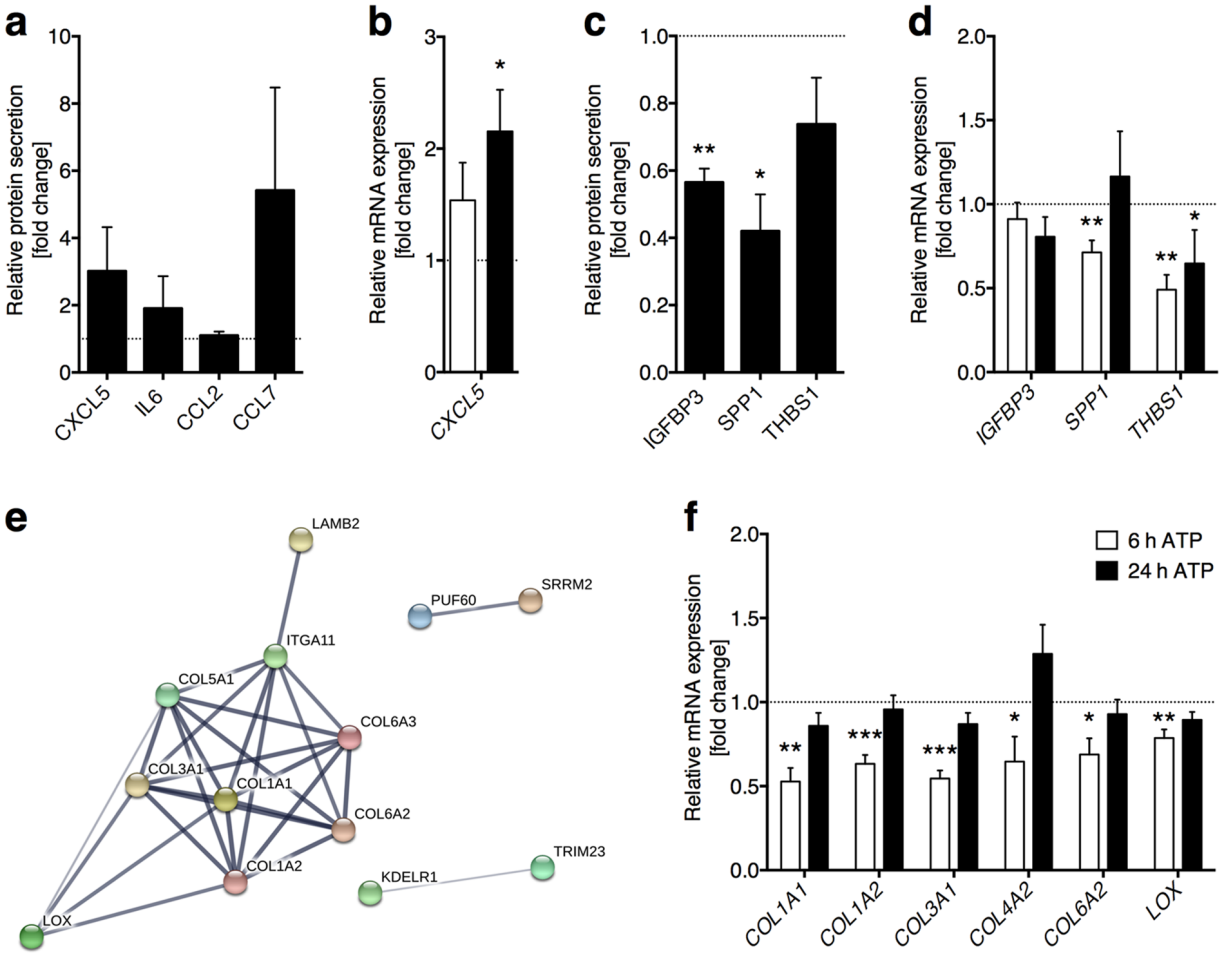
ATP treatment influences cytokine secretion and ECM protein expression. (**a**) CXCL5 and CCL7 levels were numerically, but not statistically significantly elevated after ATP treatment of HTPCs, whereas IL6 and CCL2 levels were unaltered (n = 4 each). (**b**) At mRNA level, *CXCL5* increased statistically significantly (n = 8/6). (**c**) ATP treated supernatants of HTPCs exhibited a statistically significant decrease in IGFBP3, SPP1 and a numerical decrease in THBS1 (n = 4 each) protein levels. (**d**) Decrease of *IGFBP3* (n = 8), *SPP1* (n = 8/6) and *THBS1* (n = 8) was recapitulated on mRNA level, yet only the later two reached statistical significance. (**e**) Down-regulated candidate proteins after ATP treatment clustered for enrichment in pathways or GO terms related to ECM organization and collagen metabolism. Collagens were the most highly abundant proteins within this subset. (**f**) ATP-induced decrease of collagens was confirmed by reduced mRNA levels of *COL1A1*, *COL1A2*, *COL3A1*, *COL4A2*, *COL6A2* and *LOX* (n = 8 each) after 6 h, but not 24 h of treatment. Data are means ± SEM (protein: after 48 h; mRNA: after 6 h and 24 h), normalized to control conditions. Asterisks denote statistical significance, *p < 0.05, **p < 0.01, ***p < 0.001 (one-sample *t*-test).

Cytokine profiling of ATP-treated supernatants further revealed purinergic regulation of cytokine secretion in HTPCs (Fig. 6a,c). Firstly, we analysed those cytokines previously observed on transcript level. IL1B and IL33 signals were below detection limit and IL6 and CCL2 were only marginally up-regulated. In comparison, levels of the immunosuppressive cytokines, e.g. IL3, IL4 or IL10, were not affected (not shown). By contrast, CCL7 abundance appeared numerically increased in treated HTPCs. In addition, we identified another factor, CXCL5, with elevated expression after ATP treatment, yet this increase did not reach statistical significance (Fig. 6a). *CXCL5* mRNA expression levels in ATP-treated samples were however statistically significantly augmented at 24 h (Fig. 6b). Among secreted proteins with reduced abundance after ATP treatment three previously unexpected candidates were determined: insulin-like growth factor-binding protein 3 (IGFBP3), osteopontin (SPP1) and thrombospondin 1 (THBS1), although only the first mentioned two factors were statistically significantly decreased (Fig. 6c). Here, concomitant detection of mRNA decrease reinforced our findings except for IGFBP3 (Fig. 6d).

The third method, mass spectrometry of whole cell pellets, identified 83 in abundance affected candidate proteins (*p*-value < 0.05; log2-fold change > |0.6|, Supplementary Table 1) in ATP-treated HTPCs (n = 6, 48 h), 38 were reduced in abundance and 45 were increased in abundance. All proteins that were quantified (3533 proteins, identification with two individual peptides, false discovery rate (FDR) <1%) are listed in Supplementary Table 1. Interaction annotation revealed clustering between proteins of decreased abundance (Fig. 6e, PPI enrichment *p*-value 2.61e^−11^), but not among those of increased abundance. For instance, six KEGG pathways were significantly enriched, the most prominent being ECM-receptor interaction (8 proteins, FDR 8.04e^−10^). Enriched GO terms were mostly related to collagen metabolism and ECM organization as well (Supplementary Table 1). To corroborate this finding, transcript abundances of several of the clustering proteins were compared in ATP-treated *versus* untreated HTPCs (Fig. 6f). Significant decreases in collagens *COL1A*1, *COL1A2*, *COL3A1*, *COL4A2*, *COL6A2* and lysyl oxidase (*LOX*) mRNA after 6 h corresponded to the results of the proteomic study.

All three approaches yielded different, but yet consistent results. Elevated cytokine production and secretion in response to ATP reinforce the proposition of ATP as a danger molecule able to promote (pro-)inflammatory processes in HTPCs. Meanwhile smooth muscle-like cell markers and ECM-secretive properties of the HTPCs were negatively influenced.

## Discussion

Extracellular ATP serves as a danger molecule in a variety of tissues^32^, but its role in the human testis in health and disease has been poorly examined. To explore its actions in the testicular environment of men we turned to HTPCs, to date the sole human testicular cell culture model available, which provides the possibility of mechanistic studies^6^. We applied complementary approaches and found that ATP can evoke distinct changes in HTPCs, which can foster inflammation. Therefore elevated levels of testicular ATP may contribute to male infertility.

In the testis, extracellular ATP can originate from different sources, including Sertoli cells^33^ and activated immune cells^18,34^. Strikingly increased numbers of activated mast cells have previously been reported in testes of infertile patients, specifically in the peritubular compartment^3,20^. We confirmed and correlated this finding with the expression of the purinergic receptors P2RX4 and P2RX7 in the tubular wall *in vivo* and *in vitro*. A recent study in the lung identified a link between increased extracellular ATP levels, P2RX7 expression and development of fibrosis^35^. Together, these findings may hint to a role of ATP in sterile inflammatory events associated with male infertility.

Notably, we observed inter-individual differences in P2X receptor expression. This variability may result from patient’s variety and lifestyle, which cannot be controlled for, but also from the extent of impaired testicular function. These fluctuations in expression levels may explain variations in ATP-induced gene expression and cytokine release.

Electrophysiological characteristics of cultured HTPCs stemming from two different men pointed to P2RX4 as the prevalent purinergic receptor compared to P2RX7. Measurements of intracellular [Ca^2+^] changes supported the hypothesis of a P2RX-mediated effect since ATP-induced Ca^2+^ signals depended on the presence of extracellular Ca^2+^. Both a specific P2RX7 antagonists and a high affinity blocker for P2RX1–3 and P2RX5 failed to substantially inhibit ATP-dependent [Ca^2+^] elevations, whereas micromolar concentrations of PPADS dose-dependently blocked such signals. Consistent with the electrophysiological properties *I*_ATP_ in HTPCs, our findings suggest predominant functional expression of P2RX4. However, co-assembly and co-activation with P2RX7 cannot be excluded^36,37^ and require future investigation.

To date, peritubular cells have primarily been characterized by the expression of smooth muscle cell markers^21,38^ and loss of contractile markers has been associated with infertility^39^. Hence, ATP-mediated *ACTA2* and *CNN1* decreases in HTPCs could be first steps *en route* to infertility.

CXCL12^10,40^ and GDNF^9,41^ are by HTPCs-produced and -secreted factors with importance for spermatogonial stem cell maintenance. Their mRNA levels were significantly, but not drastically altered by ATP treatment after 6 h and only marginally changed after 24 h. CXCL12 secretion (n = 4, *p* = 0.762; not shown) remained unchanged in response to ATP. Thus, ATP does not alter these contributions of HTPCs to the stem cell niche.

By contrast, prior studies of HTPCs revealed their ability to secrete immunoregulatory factors^10^. Recently, functional Toll-like receptors were identified in HTPCs and a danger signal from the ECM, biglycan, was implicated in their activation, entailing secretion of the chemo-/cytokines CCL2 and IL6^11^. ATP was also able to induce CCL2 and IL6 production and secretion in HTPCs, similar to results from other cell types^42–45^. However, IL6 and CCL2 levels were only mildly elevated. In contrast, CCL7, a sister molecule of CCL2, exhibited a strong increase in response to ATP. A comparable result has previously been reported in murine mast cells^46^. IL1B is the prototypical pro-inflammatory cytokine and highly elevated by extracellular ATP in HTPCs and other cells e.g. via purinergic mechanisms^47–49^. Its family member IL33 is constitutively expressed by smooth muscle cells^50^ and plays an important role during inflammation in diseases associated with tissue fibrosis^51^. CXCL5 is a novel player in ATP-mediated cytokine regulation, as it has mainly been known as a chemoattractant in neutrophil recruitment^52^. Yet, it has the ability to bind to the same receptor as CCL2 and CCL7 and may be jointly regulated^53,54^. Together, ATP evoked an increase in (pro-)inflammatory and immunoregulatory gene expression and corresponding protein secretion in HTPCs.

All identified factors reduced in abundance by ATP are important components of the ECM^55^. While collagens mainly form the ECM core structure, THBS1, SPP1 and IGFBP3 exhibit various interactive properties. THBS1 is able to bind to collagens and other ECM molecules, including biglycan, thereby modulating cell-matrix interactions^56^. SPP1 is expressed in numerous cell types including smooth muscle cells^57^ and is involved as pro-inflammatory factor in macrophage recruiting and cytokine secretion^58^. Moreover, THBS1 and SPP1 have been described to directly bind to IGFBP3 (albeit with lower affinity than to IGFBP5, which was not included in our secretion array)^59^. Increased IGFBP3, apart from its role in regulation of soluble insulin-like growth factor availability, can also induce elevated collagen production in smooth muscle cells^60^. This could imply the possibility of a collagen down-regulation upon a decrease in IGFBP3.

We are not aware of comparable *in vitro* studies examining ATP action on ECM in other human smooth muscle cells, yet the result of ATP-induced ECM molecule decreases in HTPCs contrast *in vivo* findings from systemic studies of both the kidney and respiratory tract in mice^61,62^. It therefore remains to be determined whether and, if so, how the ATP/P2RX4 axis of peritubular cells may be related to testicular fibrotic ECM deposits in men suffering from infertility. Such ECM deposits could conceivably develop as a consequence of an overall inflammatory environment *in vivo*. Temporal aspects may also play a crucial role. Since in our study the mRNA decrease was observed mostly at 6 h, but was restored at 24 h, only a transient decline in ECM production may occur. Limited access to patient material prevented us from further investigation.

In summary, the experimental approaches delivered complementary results, which taken together provide mechanistic insights into the actions of ATP in the human testis. Specifically, elevated cytokine production and secretion in response to ATP reinforce the proposition of ATP acting via P2X receptors as a danger molecule able to promote (pro-)inflammatory processes in HTPCs.

## Material and Methods

### Cell culture and treatment

HTPCs were isolated from human testicular tissue samples exhibiting normal spermatogenesis as described previously^21,63^. The patients (undergoing reconstructive surgery of the vas deferens, 36–52 years old) had granted written Informed Consent for the scientific use of the cells. The local Ethical Committee (Ethikkommission, Technische Universität München, Fakultät für Medizin, München, project number 5158/11) has approved the study. All experiments were performed in accordance with relevant guidelines and regulations (including all laboratory and biosafety regulations). Cells were cultivated in DMEM High Glucose (Gibco, Paisley, UK) supplemented with 10% fetal bovine serum (FBS, Capricorn Scientific, Ebsdorfergrund, Germany) and 1% penicillin/streptomycin (Biochrom, Berlin, Germany) at 37 °C, 5% (v/v) CO_2_. For the experiments defined cell numbers were seeded onto dishes or plates. 24 h prior to stimulation cells were serum starved. As agent for treatment ATP (1 mM, Sigma-Aldrich, Steinheim, Germany) was applied. Pilot studies were performed to evaluate optimal time frames for treatment depending on the applied method. Transcript levels were measured 6 h and 24 h post stimulation, whereas mass spectrometry and cytokine profiling were performed 48 h post stimulation.

### RT-PCR and qPCR

Total RNA from harvested cells was extracted using the RNeasy Plus Micro Kit (Qiagen, Hilden, Germany). Reverse transcription of 200 ng or 1 µg RNA was performed utilizing SuperScriptII (Invitrogen, Darmstadt, Germany) and random 15mer primers. For qPCR studies the QuantiFast SYBR Green PCR Kit (Qiagen, Hilden, Germany) was applied using the primers depicted in Table 1 (designed using Primer3, http://primer3.wi.mit.edu, final concentration 300–900 nM) for amplification. Samples (final cDNA concentration 2 or 20 ng/reaction) were analysed in duplicates in a LightCycler^®^ 96 System (Roche Diagnostics, Penzberg, Germany) under following conditions: Pre-incubation (95 °C, 5 min), 35–42 cycles of denaturation and annealing/extension (95 °C, 10 sec/annealing temperature see Table 1, 30 sec) followed by a melting step (continuous heating from 65 °C to 97 °C) and a cool-down (37 °C, 30 sec). Amplicon identity was confirmed via agarose gel electrophoresis and sequence analysis (GATC, Konstanz, Germany). Basal levels and therefore levels after treatment between HTPCs from different patients varied as exemplified for *IL1B* in Supplementary Fig. 4a,b. Thus, analysis of results was performed according to the 2^−ΔΔCq^ method^64^ and expression was normalized to *RPL19* and *HPRT* as endogenous reference. Reference genes exhibited stable C_q_ values in all samples measured. The corresponding boxplots and statistics are depicted in Supplementary Fig. 4c,d.

**Table 1:** Oligonucleotide primer sequences and corresponding parameters for qPCR experiments.

Gene	Reference ID	Nucleotide sequence	Amplicon size	Annealing temperature
*ACTA2*	NM_001613.2	5′-ACA ATG AGC TTC GTG TTG CC-3′ 5′-GAG TCA TTT TCT CCC GGT TGG-3′	90 bp	59 °C
*CCL2*	NM_002982.3	5′-CAG CCA GAT GCA ATC AAT GCC-3′ 5′-TGG AAT CCT GAA CCC ACT TCT-3′	190 bp	58 °C
*CCL7*	NM_006273.3	5′-TGG AGA GCT ACA GAA GGA CCA-3′ 5′-GTG GGG TCA GCA CAG ATC TC-3′	94 bp	58 °C
*CNN1*	NM_001299.5	5′-CGA AGA CGA AAG GAA ACA AGG T-3′ 5′-GCT TGG GGT CGT AGA GGT G-3′	186 bp	62 °C
*COL1A1*	NM_000088.3	5′-AAG AGG AAG GCC AAG TCG AG-3′ 5′-CAC ACG TCT CGG TCA TGG TA-3′	91 bp	60 °C
*COL1A2*	NM_000089.3	5′-CCG GAG ATA GAG GAC CAC GT-3′ 5′-CAG CAA AGT TCC CAC CGA GA-3′	132 bp	60 °C
*COL3A1*	NM_000090.3	5′-GGT GGT TTT CAG TTT AGC TAC GG-3′ 5′-TGA TGT TCT GGG AAG CTC GG-3′	106 bp	59 °C
*COL4A2*	NM_001846.3	5′-AAG GAA TCA TGG GCT TTC CT-3′ 5′-CTC TGG CAC CTT TTG CTA GG-3′	204 bp	60 °C
*COL6A2*	NM_001849.3	5′-GTC ATG AAA CAC GAA GCC TAC G-3′ 5′-CAC CCT TCT GTC CAC GGT AG-3′	97 bp	59 °C
*CXCL12*	NM_000609.6	5′-TCA GCC TGA GCT ACA GAT GC-3′ 5′-CTT TAG CTT CGG GTC AAT GC-3′	161 bp	60 °C
*CXCL5*	NM_002994.4	5′-CAG CGC TCT CTT GAC CAC TA-3′ 5′-GAA CTC CTT GCG TGG TCT GT-3′	194 bp	60 °C
*GDNF*	NM_000514.3	5′-GCA GAC CCA TCG CCT TTG AT-3′ 5′-ATC CAC ACC TTT TAG CGG AAT G-3′	93 bp	60 °C
*HPRT*	NM_000194.2	5′-CCT GGC GTC GTG ATT AGT GA-3′ 5′-GGC CTC CCA TCT CCT TCA TC-3′	163 bp	60 °C
*IGFBP3*	NM_001013398.1	5′-ACA GCC AGC GCT ACA AAG TT-3′ 5′-CTA CGG CAG GGA CCA TAT TC-3'	100 bp	59 °C
*IL1B*	NM_000576.2	5′-CTT GGT GAT GTC TGG TCC ATA TG-3′ 5′-GGC CAC AGG TAT TTT GTC ATT AC-3′	127 bp	60 °C
*IL33*	NM_001199641.1	5′-AGG TGA CGG TGT TGA TGG TAA-3′ 5′-AAG GAC AAA GAA GGC CTG GT-3′	142 bp	60 °C
*IL6*	NM_000600.4	5′-AAC CTG AAC CTT CCA AAG ATG G-3′ 5′-TCT GGC TTG TTC CTC ACT ACT-3′	159 bp	60 °C
*LOX*	NM_001317073.1	5′-CAC ACA CAC AGG GAT TGA GTC-3′ 5′-AGT CAG ATT CAG GAA CCA GGT-3′	147 bp	60 °C
*P2RX4*	NM_001256796.1	5′-AGA TGC GAC CAC TGT GTG TA-3′ 5′-GTT GAG ACT CCG TTG CTG TG-3′	78 bp	60 °C
*P2RX7*	NM_002562.5	5′-TGT CCC ATT TTC CGA CTA GG-3′ 5′-CCA ACG GTC TAG GTT GCA GT-3′	120 bp	60 °C
*RPL19*	NM_000981.3	5′-AGG CAC ATG GGC ATA GGT AA-3′ 5′-CCA TGA GAA TCC GCT TGT TT-3′	199 bp	60 °C
*SPP1*	NM_001040058.1	5′-TTT TCA CTC CAG TTG TCC CC-3′ 5′-TAC TGG ATG TCA GGT CTG CG-3′	109 bp	59 °C
*THBS1*	NM_003246.3	5′-AGT CGT CTC TGC AAC AAC CC-3′ 5′-AGC TAG TAC ACT TCA CGC CG-3′	148 bp	60 °C
*TPSAB1*	NM_003294.3	5′-GCG ATG TGG ACA ATG ATG AG-3′ 5′-CAA GGT GGT ATT TTG CGT CA-3′	102 bp	60 °C

### Immunoblotting

Whole cell lysates were generated and blotted as described previously^11,19^. The following antibodies were used: Polyclonal rabbit anti-P2RX4 IgG (1:100, HPA039494, Atlas Antibodies, Stockholm, Sweden) and monoclonal rabbit anti-P2RX7 IgG (1:500, GTX62830, GeneTex/Biozol, Eching, Germany). For detection HRP-conjugated corresponding secondary antibodies and chemiluminescent solutions (SuperSignal^®^ West Femto Maximum Sensitivity Substrate; Pierce, Thermo Scientific, Rockford, IL, USA) were applied.

### Supernatant protein profiling

Supernatants of ATP-treated *versus* untreated cells were collected and analysed using the Proteome Profiler Human XL Cytokine Array Kit (R&D Systems, Minneapolis, MN, USA) according to the manufacturer’s instructions. Quantitative analysis of spot density was performed using Fiji^65^.

### Immunohistochemistry

Immunohistochemical staining of paraffin-embedded human testicular tissue samples fixed by Bouin’s solution was performed as described previously^11^. They include samples from patients with mixed atrophy, in which tubules with normal spermatogenesis coexist with tubules bearing impaired spermatogenesis and a thickened wall. The local Ethical Committee (Ethikkommission, Technische Universität München, Fakultät für Medizin, München, project number 5158/11) has approved the study. Samples were sectioned at 5 µm thickness for immunostaining. The following antibodies were applied: Polyclonal rabbit anti-P2RX4 IgG (1:50, HPA039494, Atlas Antibodies, Stockholm, Sweden), polyclonal rabbit anti-P2RX7 IgG (1:50, HPA042013, Atlas Antibodies, Stockholm, Sweden), monoclonal mouse anti-Mast Cell Tryptase antibody (1:300, M7052, Dako, Carpinteria, CA, USA), monoclonal rabbit anti-Calponin-1 (1:250, 1806–1, Epitomics, Burlingame, CA, USA) and monoclonal mouse anti-Actin, α-Smooth Muscle antibody (1:2000, A5228, Sigma-Aldrich, St. Louis, MO, USA). Negative controls consisted of pre-adsorbed primary antibody for P2RX4 and of omission of the primary antibody for P2RX7, SMA, CNN1 and tryptase or incubation with non-immune serum instead of the antibody. Sections were counterstained with hematoxylin.

### Whole cell pellet proteome analysis

Cell lysis was performed with an ultrasonic device (10,000 kJ, Sonoplus GM3200 with BR30 cup booster, Bandelin, Berlin, Germany) in 7 µl 8 M urea/0.4 M NH_4_HCO_3_ per 100,000 cells. Samples were further centrifuged through QIA-Shredder devices (Qiagen, Hilden, Germany). Protein concentrations were determined by a Bradford assay^66^ and the samples were adjusted with 8 M urea/0.4 M NH_4_HCO_3_ to a concentration of 2 mg/ml protein. Cysteine residues were reduced for 30 min using DTE at a concentration of 4.5 mM and blocked with iodoacetamide (final concentration 10 mM) for 30 min in the dark. Samples were diluted with water to a concentration of 1 M urea and trypsinised overnight at 37 °C using 20 ng porcine trypsin (Promega, Madison, WI, USA) per µg of protein. For LC-MS/MS analysis an Ultimate 3000 chromatography system (Thermo Scientific, Waltham, MA, USA) coupled to a TripleTOF 5600+ mass spectrometer (Sciex, Concord, Canada) was used. 2.5 µg of peptides diluted in 0.1% formic acid (FA) were injected on a trap column (Acclaim PepMap 100, μ-Precolumns, 5 mm × 300 μm, 5 μm particles, Thermo Scientific) and separated at a flow rate of 200 nL/min (Acclaim PepMap RSLC C18, 75 μm × 50 cm, 2 μm; Thermo Scientific). The LC method consisted of consecutive gradients from 5% to 25% solvent B (0.1% FA, 100% ACN) in 290 min and from 25% to 50% solvent B in 30 min. MS spectra were acquired using a top 70 method (mass range m/z 400–1250, rolling collision energy activated). Peptide identification and LFQ quantification was performed with MaxQuant V1.5.1^67^ using the *Homo sapiens* subset of the UniProt database. For identification, the MaxQuant default parameters for Sciex TOF instruments were used and the FDR at the peptide and protein level was set to 1%. Student’s *t*-test was performed with the Perseus module of MaxQuant. To handle missing values the Perseus imputation feature was used in cases where proteins were detected in at least three replicates of one group. Enrichment analysis was performed with the STRING analysis tool (http://string-db.org, version 10.0) using default options^68^.

### Chemicals and solutions for (electro)physiological recordings

The following solutions were used:

(**S**_**1**_) 4-(2-Hydroxyethyl)piperazine-1-ethanesulfonic acid (HEPES) buffered extracellular solution containing (in mM) 145 NaCl, 5 KCl, 1 CaCl_2_, 0.5 MgCl_2_, 10 HEPES; pH = 7.3 (adjusted with NaOH); osmolarity = 300 mOsm (adjusted with glucose).

(**S**_**2**_) Extracellular low Ca^2+^ solution containing (in mM) 145 NaCl, 5 KCl, 1.5 CaCl_2_, 0.62 MgCl_2_, 10 HEPES, 2.37 EGTA; pH = 7.3 (NaOH); osmolarity = 300 mOsm (glucose), [Ca^2+^]_free_ = ~165 nM.

(**S**_**3**_) Standard pipette solution containing (in mM) 143 KCl, ~2 KOH, 1 EGTA, 0.3 CaCl_2_, 10 HEPES, 1 Na-GTP ([Ca^2+^]_free_ = 120 nM); pH = 7.1 (adjusted with KOH); osmolarity = 290 mOsm (glucose).

(**S**_**4**_) Cs^+^-based pipette solution containing (in mM) 143 CsCl, ~2 CsOH, 1 EGTA, 0.3 CaCl_2_, 10 HEPES, 1 Na-GTP ([Ca^2+^]_free_ = 120 nM); pH = 7.1 (adjusted with CsOH); osmolarity = 290 mOsm (glucose).

Free Ca^2+^ concentrations were calculated using WEBMAXC (http://web.stanford.edu/~cpatton/webmaxcE.htm). If not stated otherwise, chemicals were purchased from Sigma (Schnelldorf, Germany). Pyridoxal phosphate-6-azo(benzene-2,4-disulfonic acid) tetrasodium salt hydrate (PPADS; cat. no. P178) was purchased from Sigma (Schnelldorf, Germany). 3-[[5-(2,3-Dichlorophenyl)-1H-tetrazol-1-yl]methyl]pyridine hydrochloride (A-438079; cat. no. 2972) was purchased from Tocris Bioscience (Bristol, UK). 2′,3′-O-(2,4,6-Trinitrophenyl)adenosine-5′-triphosphate tetra(triethylammonium) salt (TNP-ATP; cat. no. BN0523) was purchased from BIOTREND (Cologne, Germany). Final solvent concentrations were ≤0.1%. When high ATP concentrations (≥1 mM) were used, pH was readjusted. Solutions and pharmacological agents were applied from air pressure-driven reservoirs via an 8-in-1 multi-barrel ‘perfusion pencil’ (Science Products, Hofheim, Germany). Changes in focal superfusion^69^ were software-controlled and, if required, synchronized with data acquisition by TTL input to 12 V DC solenoid valves using a TIB 14 S digital output trigger interface (HEKA Elektronik, Lambrecht/Pfalz, Germany).

### Electrophysiology

Cultured cells were transferred to the stage of an inverse microscope (DMI 4000B, Leica Microsystems, Wetzlar, Germany) equipped for phase contrast and infrared-optimized differential interference contrast (IR-DIC). Cells were observed using phase contrast objectives (HC PL FLUOTAR 10 × 0.3 NA and HCX PL FL L 63 × 0.70 NA CORR PH2; Leica Microsystems). Images were recorded using a cooled CCD-camera (DFC360FX; Leica Microsystems). Patch pipettes (5–7 MΩ) were pulled from borosilicate glass capillaries (1.50 mm OD/1.05 mm ID; Science Products) on a PC-10 micropipette puller (Narishige Instruments, Tokyo, Japan), fire-polished (MF-830 Microforge; Narishige Instruments) and filled with pipette solution (**S**_**3**_ or **S**_**4**_ depending on experimental design). An agar bridge (150 mM KCl) connected reference electrode and bath solution. An EPC-10 USB amplifier controlled by Patchmaster v2x90.1 software (HEKA Elektronik) was used for data acquisition. We monitored and compensated pipette and membrane capacitance (*C*_mem_) as well as series resistance (*R*_series_ = 16.0 ± 7.8 MΩ (mean ± SD)). Liquid junction potentials were calculated using JPCalcW software^70^ and corrected online. Signals were low-pass filtered [analog 3- and 4-pole Bessel filters (−3 dB); adjusted to^1^/_4_−^1^/_5_ of the sampling rate (10 kHz; depending on protocol)]. If not stated otherwise, holding potential (*V*_hold_) was −40 mV. All data were recorded at RT. Voltage ramp protocols are described in the Results section.

### Ca^2+^ imaging

HTPCs were grown on 30 mm glass-bottom dishes for 1 to 4 d and loaded with fura-2/AM (5 µM; 30 min; RT; **S**_**1**_). Dye-loaded cells were washed in **S**_**1**_ and transferred to the stage of an inverted microscope (Leica DMI4000B; Leica Microsystems) equipped for ratiometric live-cell imaging with a 120 W mercury short-arc reflector lamp (Leica EL6000; Leica Microsystems), a motorized fast-change filter wheel illumination system for multi-wavelength excitation (Leica Microsystems), a 12 bit 1376 × 1032 pixel cooled monochrome CCD camera (DFC 360FX; Leica Microsystems), and LASAF 2.4 imaging software (Leica Microsystems). 4–60 cells in randomly selected fields of view (890 µm × 665 µm) were illuminated sequentially at 340 nm and 387 nm (cycle time 3 s). The average pixel intensity within user-selected regions-of-interest (ROIs) was digitized and stored on a PC. Ca^2+^-dependent fluorescence signals at 510 nm were background-corrected, calculated as the f_340_/f_380_ intensity ratio, and displayed as a function of time. Categorization of HTPCs as ATP sensitive (Fig. 3) was based on standard criteria, i.e., responses were evaluated according to a ΔR_(*f340/f387)*_ > 3 × SD *R*_(baseline)_ criterion^71^. Notably, cells that exhibited spontaneous recurrent Ca^2+^ transients could not be unequivocally categorized and were therefore discarded. Thus, the percentage of ATP-sensitive HTPCs that we report here is likely to represent an underestimate.

### Data analysis

Statistical analyses of qPCR and supernatant data were obtained using GraphPad Prism 6.0 Software (GraphPad Software Inc., San Diego, CA, USA). qPCR data were analysed via a one-sample *t*-tests of −ΔΔCq values, as were cytokine levels in the supernatant via a one-sample *t*-tests of not normalized values. Electrophysiology and Ca^2+^ imaging data were obtained from independent experiments performed on at least two days with cells derived from two different patients. Individual numbers of cells/experiments (n) are denoted in the figure and/or captions. Data were analysed offline using FitMaster 2.9 (HEKA Elektronik), IGOR Pro 6.4 (WaveMetrics, Lake Oswego, OR, USA) and Excel 2013 (15.0.4779.1001; Microsoft, Seattle, WA, USA) software. Statistical analyses were performed using paired *t*-tests. If not stated otherwise, all results are presented as means ± SEM. Corresponding *p*-values that report statistical significance (≤0.05) are individually specified in the captions.

### Data Availability

Data generated and analysed during this study are included in this published article and its Supplementary Information files. The original raw datasets generated during the current study are available from the corresponding author on reasonable request.

## Electronic supplementary material

Supplementary Dataset 2

Supplementary Dataset 1
