# Supplementary material for: ATP-mediated Events in Peritubular Cells Contribute to Sterile Testicular Inflammation
Source: Sci Rep. 2018 Jan 23;8:1431. doi: 10.1038/s41598-018-19624-3 (PMC5780482; doi:10.1038/s41598-018-19624-3)
Supplement: Supplementary file 1 — Supplementary Dataset 2 [file 41598_2018_19624_MOESM1_ESM.doc]

**Supplementary Information**

**ATP-mediated Events in Peritubular Cells Contribute to Sterile Testicular Inflammation**

Lena Walenta^1^, David Fleck^2^, Thomas Fröhlich^3^, Hendrik von Eysmondt^2^, Georg J. Arnold^3^, Jennifer Spehr^2^, J. Ullrich Schwarzer^4^, Frank-Michael Köhn^5^, Marc Spehr^2^, Artur Mayerhofer^1^*

^1^ Biomedical Center Munich (BMC), Cell Biology, Anatomy III, Ludwig-Maximilians-Universität München, 82152 Planegg-Martinsried, Germany

^2^ Department of Chemosensation, Institute for Biology II, RWTH Aachen University, 52074 Aachen, Germany

^3^ Laboratory for Functional Genome Analysis LAFUGA, Gene Center, Ludwig-Maximilians-Universität München, 81377 Munich, Germany

^4^ Andrology Center, 81241 Munich, Germany

^5^ Andrologicum, 80331 Munich, Germany

*Corresponding author:

Artur Mayerhofer, MD, Biomedical Center Munich (BMC), Cell Biology, Anatomy III, Ludwig-Maximilians-Universität München, Grosshaderner Strasse 9, 82152 Martinsried, Germany

email: Mayerhofer@lrz.uni-muenchen.de

Tel.: +49 89 2180 75859

**Supplementary Material and Methods**

**Cytotoxicity assay**

Chemical compound-mediated cytotoxicity of ATP was assessed using the Pierce LDH Cytotoxicity Assay Kit (Thermo Scientific, Rockford, IL, USA) according to the manufacturer’s instructions.

**Live cell imaging**

Defined cell numbers were seeded onto a μ-Dish (35 mm, high; ibidi, Martinsried, Germany) overnight, then 24 h serum-deprived before treatment. Live cell observation for up to 72 h under incubator-like conditions was performed as described previously[^1^](#_ENREF_1). Time-lapse series were generated by taking a picture every 20 min with a ProgRes MF camera (Jenoptik, Jena, Germany) in a transmitted light microscope (Axiovert 135; Zeiss). Cell confluence and number of treated *versus* untreated cells were evaluated with Fiji (plugins: PHANTAST, cell counter).

**Immunofluorescence**

For immunofluorescence HTPCs were seeded onto cover slips, fixed in 3.7% formaldehyde (Sigma, St. Louis, MO, USA) and permeabilized with ice-cold 0.2% Triton X-100/PBS. Blocking was achieved by 0.1% Triton X-100/PBS + 5% goat normal serum (Sigma, St. Louis, MO, USA). Primary monoclonal rabbit anti-Calponin-1 antibody (1:300, 1806-1, Epitomics, Burlingame, CA, USA) was applied, as secondary antibody goat anti-rabbit Alexa Fluo 488 (Thermo Fisher Scientific, Waltham, MA, USA) was used. Cells were counterstained with DAPI and mounted for imaging. Negative controls consisted of rabbit IgG or normal goat serum instead of primary antibody.

**Data analysis**

Statistical analyses of cell viability were obtained using GraphPad Prism 6.0 Software (GraphPad Software Inc., San Diego, CA, USA). To examine differences in confluence or cell numbers paired *t*-tests (two-tailed) were applied. Analysis of cytotoxicity extent was performed with a one-sample *t*-test. Individual numbers of cells / experiments (n) are denoted in the figure and / or captions. Corresponding *p*-values that report statistical significance (≤ 0.05) are individually specified in the captions.

**Supplementary Figure 1. Calponin expression in cultured HTPCs.**

Virtually all cultured HTPCs expressed calponin (**a**,**b**; depicted in green), a typical smooth muscle marker as determined by immunofluorescence. Nuclei were visualized by DAPI staining (depicted in blue). Negative controls: Rabbit IgG (inset in **a**, **c**), omission of primary antibody (**d**). Bars = 25 µm.

**Supplementary Figure 2. P2RX4 and P2RX7 and mast cells in the tubular wall.**

P2RX4 expression (**a**) was detected in peritubular cells, germ cells and interstitial areas. P2RX7 expression (**b**) was confined to peritubular cells. Magnitude of both P2RX4 and P2RX7 expression appeared to be associated with fibrotically altered tubular wall thickness (arrows). Insets: Negative controls (pre-adsorption for P2RX4, omission of primary antibody for P2RX7); Bars = 20 µm. (**c**-**f**) Expression of P2RX7 in the tubular wall (**c**,**e**) appeared in close proximity to tryptase-stained mast cells (**d**,**f**), the probable source of extracellular ATP near the peritubular cells. Bars = 25 µm.

**Supplementary Figure 3. ATP treatment did not alter cell viability.**

Viability was assessed by total cell number (**a**, n = 4), cell confluence determination (**b**, n = 4) and cytotoxicity measurement via extracellular LDH activity (**c**, n = 3) at chosen time points over a period of 72 h. ATP stimulation did not exhibit a negative effect in comparison to untreated cells. ATP-treated cells proliferated slightly more than untreated cells (albeit significantly at 6 h) and confluence was also slightly elevated, whereas cytotoxicity decreased.

Data are means ± SEM normalized to start conditions. Asterisks (*) denote statistical significance, *p* < 0.05 (cell counts, confluence: paired *t*-test, cytotoxicity: one-sample *t*‑test).

**Supplementary Figure 4. ATP induced elevation of *IL1B* in different HTPCs and stability of reference genes C_q_ values.**

*IL1B* expression was elevated in all HTPC isolations after 6 h (**a**) and 24 h (**b**) in ATP-treated *versus* control samples. Note that basal (control) levels vary among cells from individual patients. Data depict averaged -ΔC_q_ values of *IL1B* compared to reference genes *RPL19* and *HPRT*. Boxplot (**c**) and statistical analyses (**d**) of raw C_q_ values for *RPL19* and *HPRT* over all examined HTPC samples showed low variation between samples. Therefore, reference gene expression was assumed stable in HTPCs.

**Supplementary Figure 5. Original gel and blot pictures.**

Original pictures of agarose gels for (**a**) *P2RX4*, (**b**) *P2RX7*, (**c**) *ACTA2*, (**d**) *CNN1* (**e**) *TPSAB1*, that were cropped for Fig. 1a. Cropped parts are marked by red rectangles. m: Thermo Scientific GeneRuler Low Range DNA Ladder, +: human testis 1-4: individual HTPCs, h: human mast cells (HMC-1), -RT: non-reverse transcription control, –: non-template control. Amplicon length and relevant ladder fragments are denoted in [bp].

Original pictures from Western blots for (**f**) P2RX4 and (**g**) P2RX7 that were cropped for Fig. 1b. Cropped parts are marked by red rectangles. M: Thermo Scientific PageRuler Plus Prestained Protein Ladder, a-c: individual HTPCs. Ladder weights are denoted in [kDa].

**References**

1 Windschuttl, S. *et al.* Are testicular mast cells involved in the regulation of germ cells in man? *Andrology* **2,** 615-622, doi:10.1111/j.2047-2927.2014.00227.x (2014).

2 Schindelin, J. *et al.* Fiji: an open-source platform for biological-image analysis. *Nat Methods* **9,** 676-682, doi:10.1038/nmeth.2019 (2012).

3 Jaccard, N. *et al.* Automated method for the rapid and precise estimation of adherent cell culture characteristics from phase contrast microscopy images. *Biotechnol Bioeng* **111,** 504-517, doi:10.1002/bit.25115 (2014).
